# Conservatively Managed Grade V Blunt Renal Injury

**DOI:** 10.7759/cureus.97369

**Published:** 2025-11-20

**Authors:** Nikita Rana, Ashwini Prakash

**Affiliations:** 1 Medical School, University College London, London, GBR; 2 Medicine and Surgery, Wexham Park Hospital, Slough, GBR; 3 Oncology, Royal Berkshire Hospital, Reading, GBR

**Keywords:** angioembolization, conservative management, grade v renal injury, hypotension, operative management, road traffic accident

## Abstract

Renal trauma is a relatively common clinical entity encompassing a broad range of presentations and etiologies. High-grade renal injuries represent the most severe end of this spectrum and are associated with substantial immediate and long-term morbidity and mortality. Accurate triage and timely management are therefore crucial for optimizing patient outcomes. We report the case of a 26-year-old otherwise healthy male who sustained a Grade V renal injury following a road traffic accident. The injury was identified through a primary trauma survey and was managed conservatively despite clinical indicators favoring operative intervention. Unusual features of this case included presentation with isolated right-sided chest pain, imaging evidence of significant renal hilum disruption with preserved renal function, absence of hematuria or urinary extravasation, and persistent hypotension despite adequate resuscitative and maintenance intravenous fluid therapy in a non-septic patient with stable interval imaging. Remarkably, the patient made a complete recovery under conservative management. This case underscores the criticality of early imaging in detecting occult renal trauma, often first recognized through primary trauma surveys in the emergency department. It highlights the nuanced decision-making process between operative and nonoperative management in high-grade renal injuries, an area where standardized clinical guidelines remain limited. Furthermore, the growing role of interventional radiology in the conservative management of renal trauma is discussed. This report emphasizes the necessity for robust follow-up protocols, including interval imaging, to facilitate early detection of complications and improve long-term outcomes in patients with high-grade renal injuries.

## Introduction

Renal trauma accounts for approximately 5% of trauma cases and 24% of abdominal solid organ injuries [1]. Most cases result from blunt trauma in the context of road traffic accidents, falls, or sports-related injuries, although penetrating injuries such as gunshot or stab wounds may also occur [2]. Renal injuries can occur in isolation or alongside other intra-abdominal visceral injuries, often depending on the mechanism of trauma [3]. Clinical signs of renal trauma include flank tenderness, ecchymosis, a palpable mass, shock, and flank pain [3,4]. Although hematuria occurs in up to 95% of cases, it may be absent in ureteral tears, vascular pedicle injuries, or pelviureteric junction (PUJ) disruption due to intra-abdominal urine extravasation [5,6].

The American Association for Surgery of Trauma Organ Injury Scale (AAST OIS) categorizes renal trauma from Grades I-V. This scale is based on vascular injuries visualized on computed tomography (CT) [6]. Grade V renal injuries (the most severe end of the spectrum) are characterized by a shattered kidney with active bleeding, extensive parenchymal loss, laceration or avulsion of the renal artery or vein, hilar devascularization, and PUJ disruption [6,7]. While low-grade renal injuries are managed conservatively with good outcomes, the management of Grade V injuries remains debated, and indicators for operative versus nonoperative management are ill-defined [1,3]. CT supports diagnosis, grading, and management, but high-grade injuries require integration of clinical and biochemical markers, which may reveal a different overall picture than imaging alone [3].

Typically, high-grade renal injuries carry increased morbidity and mortality due to hemorrhage, urinary extravasation, and long-term renal dysfunction, often presenting with hemodynamic instability [4,8]. Complications may appear within two weeks post injury and include bacterial infection requiring surgical debridement, urine leak necessitating operative intervention, delayed hemorrhage from arteriovenous malformations, and hydronephrosis [2,4]. Longer-term complications can include chronic pyelonephritis and post-renal hypertension [2,4].

Operative intervention has historically been the standard for high-grade renal trauma. Advances in imaging, resuscitation, and interventional radiology have enabled selective nonoperative management under careful clinical supervision. Absolute indications for surgery include hemodynamic instability, failure to respond to aggressive resuscitation, and major vascular injury [3]. Relative indications for surgery include large renal pelvic lacerations, PUJ avulsion, and persistent urine leak [3]. Both operative and nonoperative strategies carry risks: surgery may result in wound infection, perinephric abscess formation, or urinary tract infection, whereas conservative management may be complicated by hematuria, acute kidney injury, urinoma formation, or delayed hemorrhage [4]. Consequently, management must be individualized, incorporating clinical status, imaging findings, and available institutional resources, with close follow-up to optimize renal preservation and long-term outcomes. In our case, the patient was successfully managed conservatively despite persistent hypotension, illustrating the potential viability of nonoperative strategies in carefully selected patients.

## Case presentation

A 26-year-old man with no past medical history presented to the Emergency Department with right-sided pleuritic chest pain that developed a few hours after a road traffic accident. The accident involved a car driving into the rear end of his bicycle during his commute to work. In consequence, he flew off the bicycle and landed on the pavement. Consciousness was lost momentarily, and superficial skin injuries were sustained. There were no other visible injuries, specific pains, nausea, or vomiting on or directly after the impact. No immediate medical attention was sought.

On examination, the abdomen was soft with marked tenderness localized to the right flank. The chest was clear on auscultation with equal air entry bilaterally and no focal rib tenderness. There was no increased work of breathing, and oxygen saturations were maintained on room air. Other clinical observations indicated that the patient was afebrile and hypotensive, with a blood pressure of 96/62 mmHg. This blood pressure is lower than the normotensive range expected for his body mass index of 26.2 kg/m², in which a systolic pressure between 115 mmHg and 135 mmHg and a diastolic pressure between 73 mmHg and 87 mmHg are generally considered normal [9]. The patient was alert and oriented to time, place, and person, with a Glasgow Coma Scale (GCS) score of 15 [10]. Apart from right-sided pain along the inferior aspect of the chest, no additional symptoms were reported. Notably, there was no visible or microscopic hematuria, which is often seen in renal trauma.

**Figure 1:**
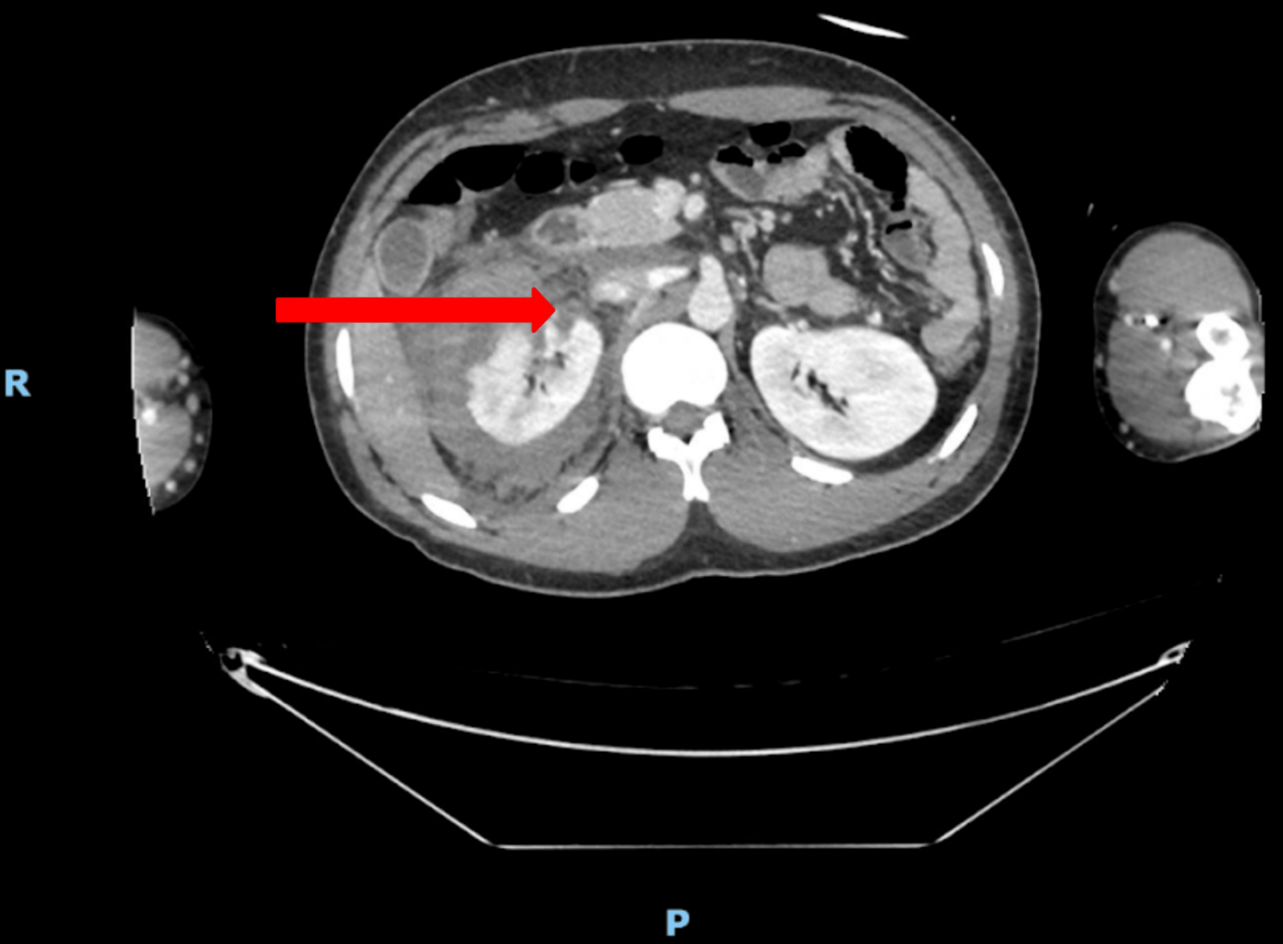
CT whole body trauma taken at hospital admission (day 0) The red arrow points to the disrupted renal pelvis in the shattered right kidney. The blue arrow points to the shattered right kidney. The letter R depicts the right-hand-side of the patient. The letter P depicts the posterior surface of the patient.

Initial investigations included venous blood gas analysis, which demonstrated an elevated lactate of 3.3 mmol/L (reference range: 0.0-1.0 mmol/L). A CT whole body was subsequently performed as part of the primary trauma survey (Figure 1). The imaging revealed a shattered right kidney with active bleeding secondary to avulsion of the renal hilum. A large hematoma was present within the right renal parenchyma, perinephric tissue, and retroperitoneal space. Based on these findings, the injury was classified as Grade V renal trauma according to the AAST scale (Table 1), and occurred in isolation as all other intra-abdominal organs appeared normal [6]. Despite the patient’s initial presentation with chest pain, the CT thorax demonstrated no evidence of acute or chronic pathology.

**Table 1:** American Association for Surgery of Trauma Organ Injury Scale The table depicts the five Grades (I-V) of renal injury according to a standardized grading system. One item from the criteria for each grade needs to be fulfilled in order for the injury to be classified as such. Source: Ref [6]

Classification	Criteria
Grade I	Subcapsular hematoma and/or contusion. No laceration
Grade II	Superficial laceration ≤1cm depth not involving the collecting system (no evidence of urine extravasation). Perirenal hematoma confined within the perirenal fascia
Grade III	Laceration >1cm not involving the collecting system (no evidence of urine extravasation). Vascular injury of active bleeding confined within the perirenal fascia
Grade IV	Laceration involving the collecting system with urinary extravasation. Laceration of the renal pelvis and/or complete ureteropelvic disruption. Vascular injury to segmental renal artery or vein. Segmental infarctions without associated active bleeding (i.e. due to vessel thrombosis). Active bleeding extending beyond the perirenal fascia (i.e. into the retroperitoneum or peritoneum)
Grade V	Shattered kidney. Avulsion of renal hilum or laceration of the main renal artery or vein: devascularization of a kidney due to hilar injury. Devascularized kidney with active bleeding

The patient was admitted under Urology for close observation, analgesia, and intravenous fluid resuscitation. An urgent group and save was sent, four units of cross-matched blood were sourced, and the patient was made nil by mouth in anticipation of surgical management. It is important to note that, during his hospitalization, the patient remained hypotensive despite resuscitation with intravenous crystalloids. Hypotension in the context of Grade V renal trauma is usually an indication for immediate operative management [3]. In this case, though surgical management was anticipated, the patient was not taken to surgery on initial presentation as his blood pressure, although hypotensive, did not drop further. There was no need for resuscitation with blood products as his hemoglobin remained stable, lactate normalized, and other vital signs (including heart rate) were within normal range. All of these factors suggested an element of stability to the renal injury with adequate tissue perfusion despite damage to the renal artery. The patient’s blood pressure was monitored closely, and there was a low threshold for surgical management should further hypotension ensue.

**Figure 2:**
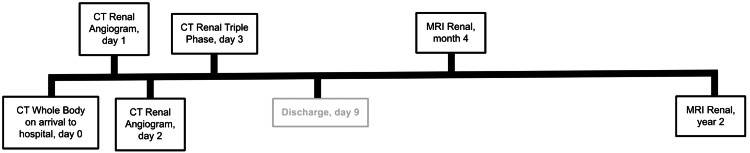
Timeline diagram of imaging Timeline diagram demonstrating the various imaging modalities listed in chronological order. The day number from the initial insult is also listed for further clarity.

The next day, Urology continued with nonoperative management due to the stability of the patient’s clinical and biochemical markers (sustained hypotension, normal renal function, and stable hemoglobin). Supportive care consisted of ongoing intravenous fluid administration and strict bed rest to minimise the risk of hematoma expansion. Parameters were assessed on a daily basis in order to gauge the stability of the renal injury, allowing clinicians to make informed decisions on whether to continue with conservative management or escalate to surgical management. Noted parameters included urine output, hourly monitoring of all vitals, serial assessment of renal function, hemoglobin levels, and inflammatory markers through daily blood tests and interval renal imaging, as shown in Figure 2. 

Furthermore, measures were taken to ensure patient safety due to the risk of deterioration. The patient was situated in an escalation bay with a contingency plan for transfer to a high-acuity unit with vasopressor support if further signs of hemodynamic instability emerged. Pharmacological venous thromboembolism prophylaxis was withheld due to the elevated bleeding risk posed by the renal injury and potential need for urgent surgery.

**Figure 3:**
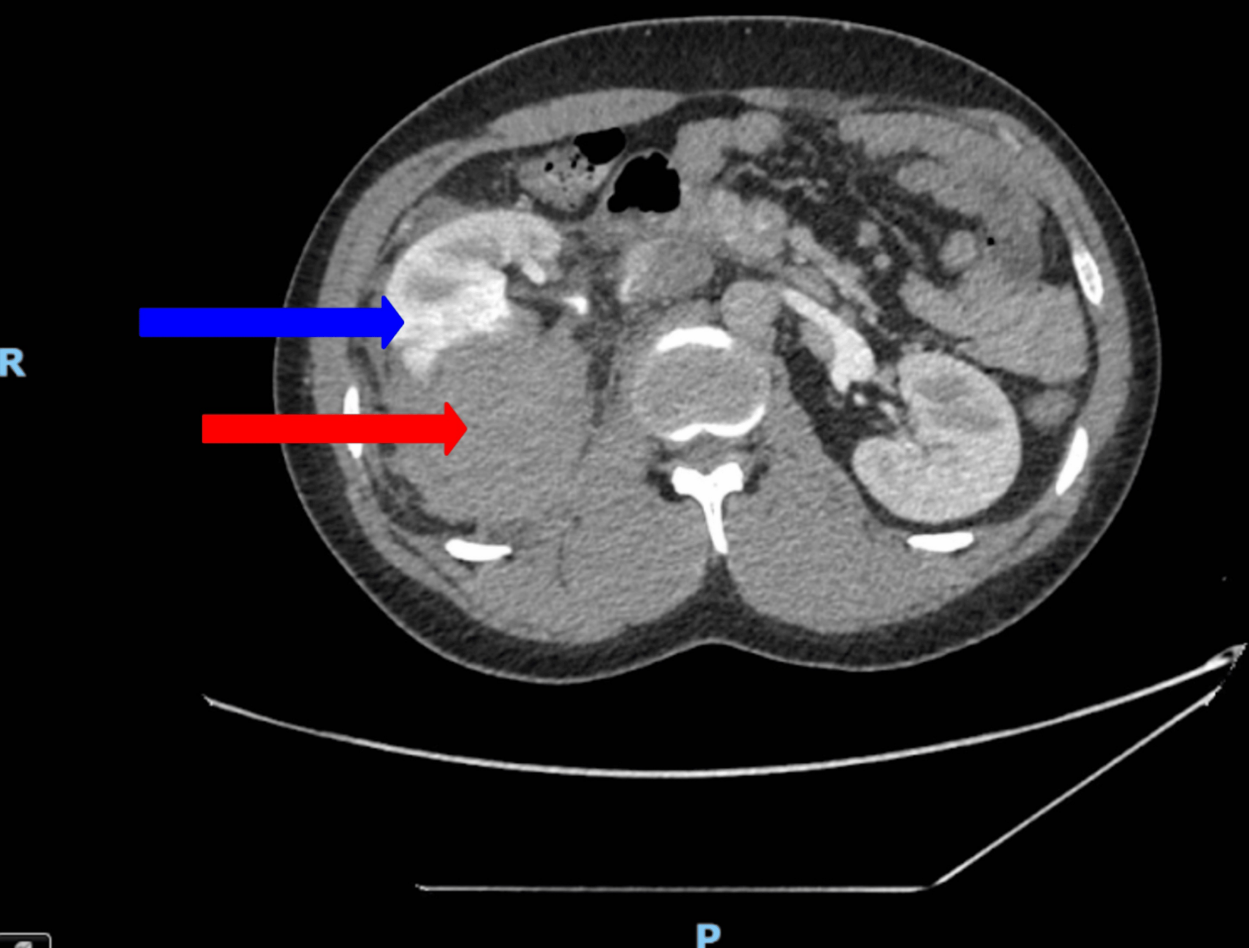
CT renal angiogram taken on day one of hospital admission The red arrow points to the stable perinephric hematoma. The blue arrow points to the shattered right kidney. The letter R depicts the right-hand-side of the patient. The letter P depicts the posterior surface of the patient.

**Figure 4:**
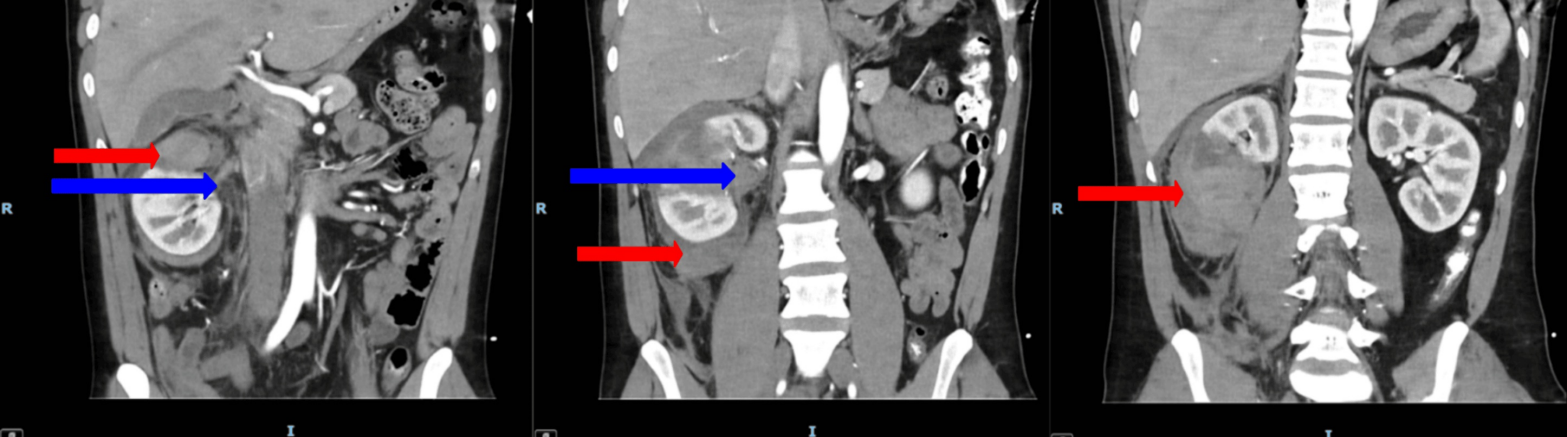
CT angiogram renal (coronal view, series) on day two of hospital admission The red arrows point to the perinephric hematoma. The blue arrows point to renal hilum disruption. The letter R depicts the right-hand-side of the patient. The letter I depicts the inferior aspect of the patient.

**Figure 5:**
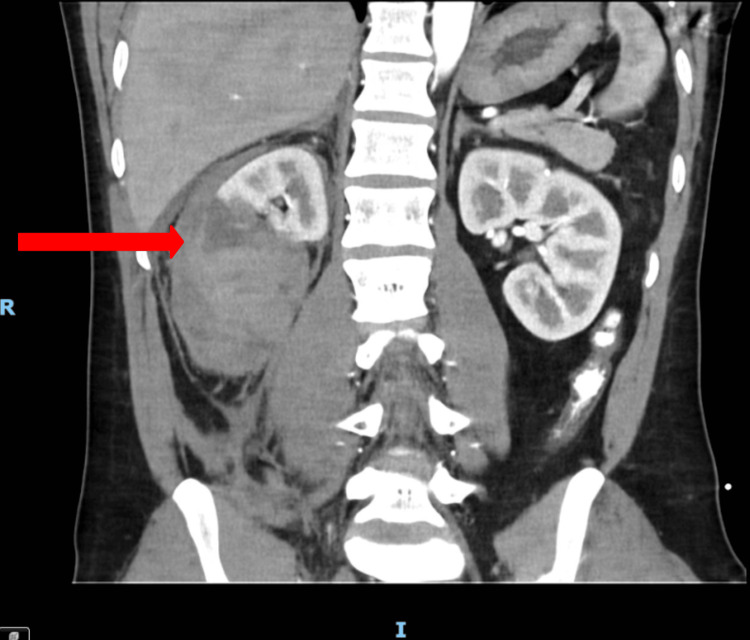
CT kidney triple phase (coronal view) taken on day three of hospital admission The red arrow points to the perinephric hematoma. The letter R depicts the right-hand-side of the patient. The letter I depicts the inferior aspect of the patient.

The extent of renal injury was assessed with regular interval imaging during the nine-day admission and after discharge, as illustrated in Figure 2. On the first day of admission, a CT renal angiogram demonstrated a stable, large right perinephric hematoma with no evidence of urinary extravasation (Figure 3). Inflammatory markers revealed a normal white blood cell (WBC) count and a C-reactive protein (CRP) level of 7.8 mg/dL (reference range: 0.0-5.0 mg/dL). On the second day, a repeat CT angiogram showed a stable renal injury (Figure 4). The WBC count remained within normal limits, but the CRP increased to 90 mg/dL (reference range: 0.0-5.0 mg/dL), prompting initiation of a six-day course of broad-spectrum intravenous antibiotics (amoxicillin-clavulanic acid). On the third day, a CT triple-phase renal demonstrated stable injuries to the right kidney (Figure 5). All imaging up until this point showed avulsion of the renal hilum and vasculature with an associated large hematoma and transected midportion, consistent with a stable Grade V renal injury. Remarkably, despite disruption to his renal pelvis, the patient’s renal function and urine output remained preserved and optimal, suggesting the possibility of an intact ureter. There were no significant changes in his vital signs, and the overall clinical picture was not suggestive of sepsis despite an isolated rise in CRP. CRP is a non-specific marker of inflammation, which may have risen in the context of kidney injury as opposed to infection.

After his fourth day in hospital, the patient was advised that he could start light ambulation guided by physiotherapists, and the previous instructions for strict bed rest were lifted. The patient remained stable, and his pain gradually improved. He was discussed in a multidisciplinary Urology-Radiology meeting. After review of his imaging, it was decided that nonoperative management would continue due to the stability of his hematoma and clinical observations. On discharge, the patient would need magnetic resonance imaging (MRI) and an outpatient appointment with Urology for clinical re-review and discussion of imaging and to plan further management.

Nine days after admission, he reported no pain, successfully passed a trial without a catheter, completed a course of broad-spectrum antibiotics, and was subsequently discharged.

**Figure 6:**
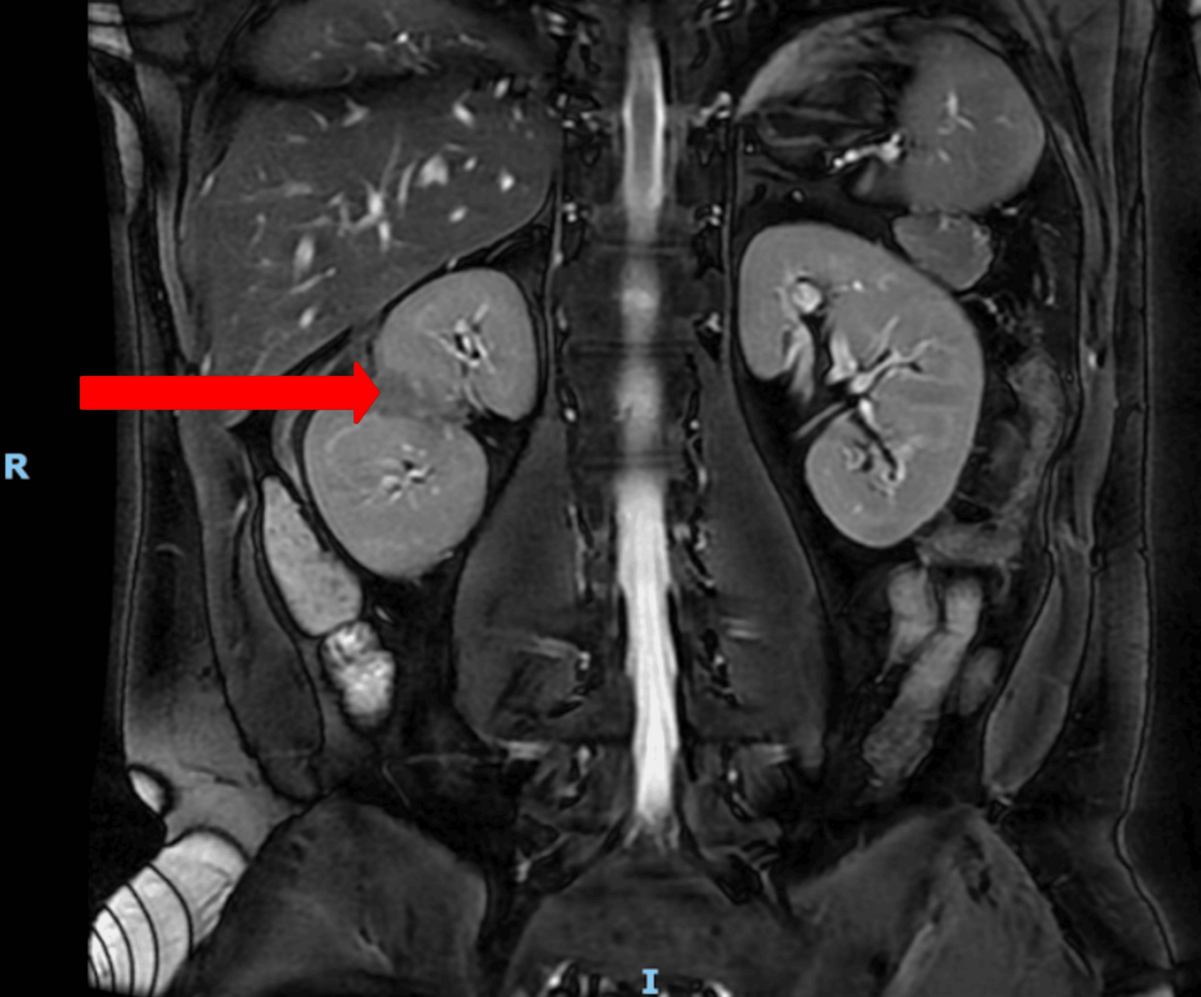
MRI renal (coronal view) taken four months after initial insult The red arrow points to cortical scarring in the interpolar region of the right kidney. The letter R depicts the right-hand-side of the patient. The letter I depicts the inferior aspect of the patient.

Four months later, he was followed up with an MRI renal. The scan showed scarring in the interpolar region of the renal capsule and the cortex of the right kidney (Figure 6). A marked improvement in the Grade V renal injury was seen in comparison to previous scans. Despite his prior low blood pressure, routine observations taken during this period of imaging demonstrated that the patient had since become normotensive.

**Figure 7:**
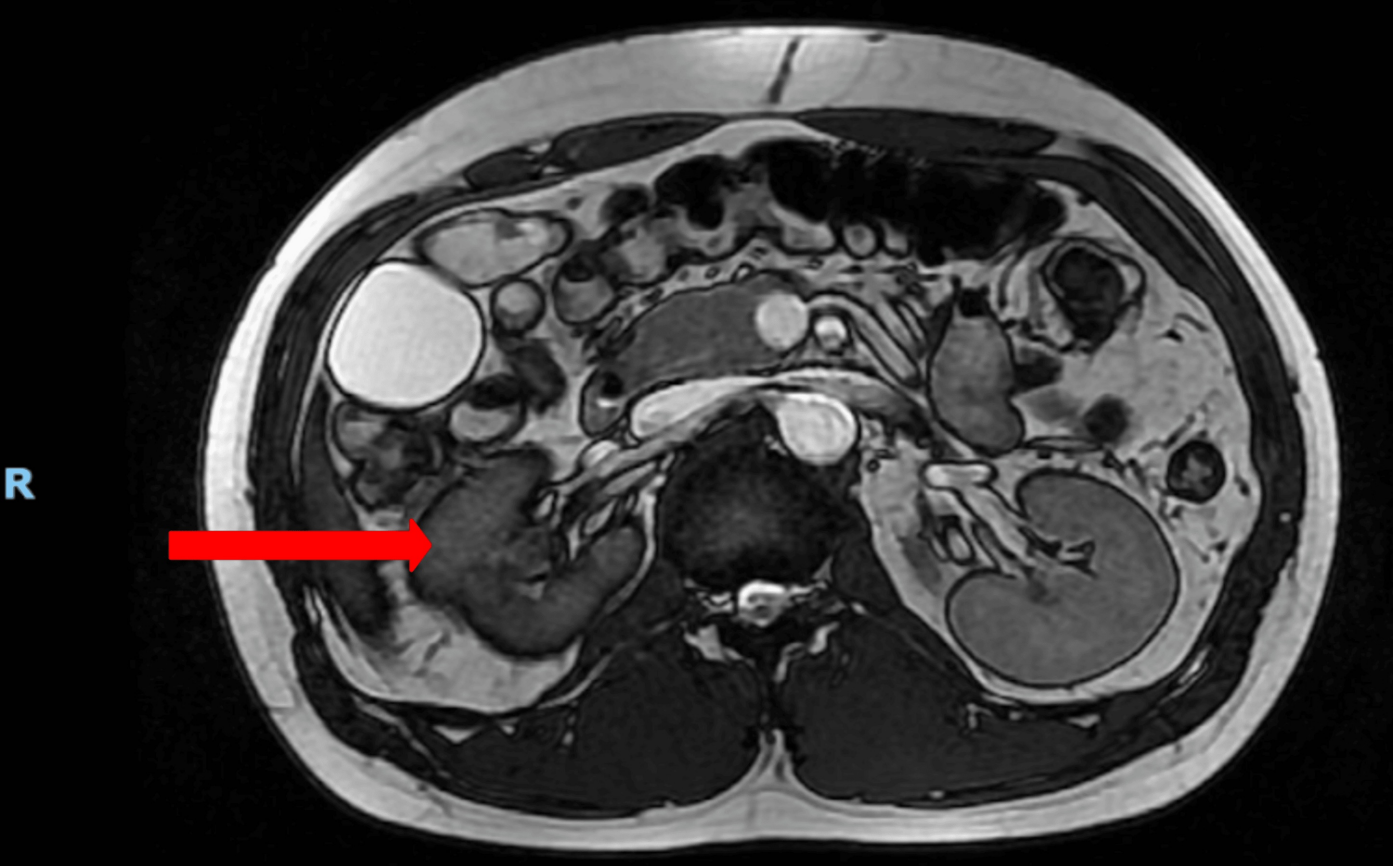
MRI renal (sagittal view) taken two years after initial insult The red arrow points to a resolved perinephric hematoma of the right kidney. The letter R depicts the right-hand-side of the patient.

The patient was unfortunately never seen in a Urology clinic; however, his MRI was reviewed in a Radiology meeting two years after initial injury. A further MRI renal was planned to assess any progressive changes to the right kidney over the two-year period. This scan showed residual scarring in the interpolar region of the right kidney but a completely resolved hematoma (Figure 7). Conservative management was deemed successful, and no further follow-up was required.

## Discussion

Grade V renal injuries account for 6-7% of all renal injuries and represent the most severe type, carrying a high risk of mortality and morbidity [3,8]. Selecting the appropriate management is paramount, as mortality rates for Grade V injuries remain 22.8% for operative management and 23% for nonoperative management [11]. These rates are broadly comparable, which led us to consider factors that may favor one approach over the other.

There are no clear or universal guidelines for the management of high-grade renal injuries, leaving management decisions largely at the discretion of the clinicians involved [1]. Factors favoring operative management include hemodynamic instability unresponsive to resuscitative measures (intravenous fluid boluses and maintenance), active bleeding from a focal vascular point, an expanding or pulsatile renal hematoma, persistent or worsening urine extravasation, and renal hilum or proximal ureteral avulsion identified on imaging [3,4]. According to the literature, persistent hypotension in this case may have favored an operative approach, although we note that no clear blood pressure cutoffs exist to guide clinicians’ evaluation in Grade V renal trauma. This case demonstrates successful conservative management despite the presence of sustained hypotension - typically considered an indicator for surgical intervention. These findings highlight the importance of comprehensively evaluating clinical and biochemical markers, alongside interval imaging, when determining the most appropriate management strategy for each individual patient with high-grade renal trauma.

An increasingly popular method for managing high-grade renal injuries is selective angioembolization. This is considered a nonoperative approach and has shown favorable patient outcomes, with successful embolization rates of up to 88% and renal salvage rates of 92% in renal trauma cases [4]. In comparison to explorative surgery, angioembolization demonstrates higher success rates for salvaging the kidney with fewer post-procedural complications. Risks reduced with this interventional radiology approach include external bleeding, infection, and general anesthetic (as angioembolization can be performed under local anesthesia) [10]. Despite the success of angioembolization, there are no validated criteria to select patients for this intervention [12]. Considerations for this approach include extravasation of contrast and expanding perirenal collections greater than 25 mm on imaging [12]. Angioembolization was not considered in this case due to the stability of the renal hematoma, and no contrast leak was visualized on imaging, as illustrated in Figure 4.

Transitioning away from nonoperative management, renal exploration via a transperitoneal approach may be indicated in selected cases based on vital signs and an elevated blood lactate level greater than 4 mmol/L (reference range: 0.0-1.0 mmol/L) [12]. The primary objectives of renal exploration include vascular control - achieved by clamping and isolating the renal artery or vein - and renal preservation through renorrhaphy or partial nephrectomy [3,12]. Total nephrectomy is reserved as a last resort for cases of extensive parenchymal destruction. If a stable perirenal hematoma is encountered intraoperatively, it should not be disturbed due to risks of uncontrolled hemorrhage and hypovolemic shock [12]. In contrast, central or expanding hematomas may suggest injury to major vessels such as the aorta or inferior vena cava, necessitating further exploration [12].

Literature suggests that, in this case, renal exploration should have been the management of choice based on the degree of injury and hemodynamic instability [12]. The reason that renal exploration was not performed remains multifactorial and reflects the extensive clinical expertise of the consulting surgeons involved. It is possible that pattern recognition, in combination with objective measures - such as low National Early Warning scores (NEWS), stable renal function and hemoglobin levels, sustained yet stable hypotension, and effective pain control - guided this decision [13]. A validated scoring tool for more clearly defined decision-making may benefit junior clinicians who have not yet developed this level of clinical intuition. An additional factor, which may have guided their choice, is the stability of the hematoma on imaging. Surgical exploration in this context may risk disruption of the hematoma, though a transperitoneal approach is often used to avoid this.

Interestingly, despite extensive renal parenchymal injury - including damage to the PUJ as demonstrated in Figure 7 - the patient’s renal function remained preserved and urine output was maintained throughout hospitalization. Furthermore, there was no evidence of urinary extravasation on CT imaging. These findings suggest that the continuity between the right renal pelvis and ureter remained functionally intact despite radiologic evidence of damage at this anatomical point. Alternatively, preserved renal function and adequate urine output may have been sustained by the function of the contralateral kidney, in which case disruption of the right PUJ would be plausible.

This case highlights that additional factors influencing management could include trends in renal function on laboratory investigations, hemodynamic response to intravenous fluid resuscitation, and changes in GCS scoring [10]. A stable estimated glomerular filtration rate and a normal GCS score of 15 were evident throughout this case despite the severity of the patient’s injuries. These two factors may serve as potential predictors of successful conservative management.

The patient in this case was a young, healthy Caucasian male involved in an urban road traffic accident. Renal trauma is typically seen in this patient population, with men accounting for 72-93% of cases and the mean age ranging from 31 to 38 years [3]. It is worth considering whether different baseline characteristics - such as comorbidities, age, or sex - would alter the management approach or threshold for operative versus nonoperative treatment. Other factors for consideration may include pre-existing kidney disease, a solitary kidney, comorbidities, advanced age, time from injury to hospital presentation, and locally available healthcare resources and expertise. Any standardized guidelines for managing Grade V renal injuries should account for varying patient factors and institutional resources.

In this case, interval imaging was critical to assess the stability, progression, or resolution of the renal injury. The patient was eventually lost to follow-up, possibly due to a lack of standardized or recommended interval imaging. The case was coincidentally discussed two years later in a Radiology meeting, where the patient was identified and recalled for follow-up. Subsequent imaging demonstrated a complete recovery of the Grade V renal injury with conservative management. Although the patient in this instance achieved full recovery, due to potential dangers posed by such severe renal trauma (including delayed bleeding risks), more formalized and robust systems for follow-up with standardized interval imaging need to be implemented. Delayed bleeding risks are most prevalent two to three weeks following initial insult, and hence imaging at this point may offer diagnostic benefit and earlier intervention for patients [4].

Renal trauma presents with a broad range of symptoms, which can make diagnosis and subsequent management challenging. Common symptoms include hematuria, abdominal pain, and a palpable renal mass [4]. Our case is unusual because chest pain was the only presenting symptom, and hematuria was absent, so renal injury was not initially considered in the Emergency Department. A Grade V renal injury was incidentally identified on a CT whole body (Figure 1), which was obtained because the symptoms could not be clinically correlated with any specific injury. This case, therefore, highlights the continued importance of performing primary trauma surveys and utilizing imaging to identify more subtle or atypical presentations of renal trauma [14]. Occult renal injury is often first identified on imaging but is occasionally more promptly identified clinically in patients with more apparent symptoms.

## Conclusions

Albeit rare, Grade V renal trauma is a serious injury necessitating immediate triage and implementation of appropriate management to reduce morbidity and mortality. Out of the available approaches, the success of unconventional conservative management, despite sustained hypotension in this patient, supports a growing shift towards a conservative approach in high-grade renal trauma cases. A lack of clear guidelines for operative versus nonoperative approaches poses a challenge to clinicians, who are increasingly opting for angioembolization as a minimally invasive yet effective way to salvage an affected kidney while negating more generic traditional operative risks. The success of conservative management in this case puts into question the degree of resuscitative measures that may be appropriate in hypotensive patients with stable renal injuries; it encourages us to move away from a tick-box approach to prescribe intravenous fluid therapies at certain blood pressure cutoffs. Additional patient and environmental factors bear significant consideration when choosing the most appropriate management, and a scoring tool to support this decision-making may be helpful. Interval imaging, including a few weeks post initial insult, is of paramount importance for monitoring purposes to promptly identify complications around re-bleeding, which may pose a serious risk to life.

We propose that a validated scoring tool to support objective decision-making by clinicians for operative versus nonoperative management of Grade V renal injuries would be beneficial in clinical practice. We also suggest that clear guidelines be published on how to manage Grade V renal injuries, taking into account data from this scoring tool. As a final reminder, we emphasize that not all high-grade renal trauma cases present timely or typically in terms of their symptom profile, so it is important to consider and actively look for renal trauma in cases of high-impact injury to avoid missing such a crucial and life-threatening diagnosis.
